# Effect of Exogenous Glycine Betaine on the Germination of Tomato Seeds under Cold Stress

**DOI:** 10.3390/ijms231810474

**Published:** 2022-09-09

**Authors:** Yingying Zhang, Taoyu Dai, Yahui Liu, Jinyan Wang, Quanhua Wang, Weimin Zhu

**Affiliations:** 1 Shanghai Key Laboratory of Protected Horticulture Technology, The Protected Horticulture Institute, Shanghai Academy of Agricultural Sciences, Shanghai 201403, China; 2 College of Life Science, Shanghai Normal University, Shanghai 201400, China; 3 Innovation Center of Jiangsu, Academy of Agricultural Sciences, Nanjing 210014, China

**Keywords:** cold stress, endogenous hormone, germination, glycine betaine (GB), tomato (*Solanum lycopersicum*), seed

## Abstract

Cold stress is known to influence tomato growth, development, and yield. In this study, we analyzed the germination of tomato seeds treated with exogenous glycine betaine (GB) at a low temperature (14 °C). The results showed that cold stress inhibited tomato seed germination, and pretreatment with exogenous GB reduced this inhibition and enhanced the germination rate (GR), germination index (GI), and viability of tomato seeds at low temperatures. Analysis of gene expression and metabolism revealed that GB positively regulated endogenous hormone gibberellin (GA) content and negatively regulated abscisic acid (ABA) content, while GB reduced the starch content in the seeds by up-regulating the amylase gene expression. Gene expression analysis showed that the key genes (*SlSOD*, *SlPOD,* and *SlchlAPX*) involved in reactive oxygen species (ROS) scavenging systems were up-regulated in GB-pretreated tomato seeds compared with the control. At the same time, levels of malondialdehyde and hydrogen peroxide were significantly lower, while the proline content and peroxidase (POD), superoxide dismutase (SOD), and catalase (CAT) levels were elevated compared with those in the control. These results demonstrate that exogenous GB as a positive regulator effectively alleviated the inhibition of tomato seed germination under cold stress by different signal pathways.

## 1. Introduction

Plants live in a constantly changing environment and are often subjected to various abiotic stresses, such as extreme temperature (cold and heat), drought, and salinity, which can seriously affect crop yield and quality [1,2,3,4]. Plants have evolved complex regulatory mechanisms to sense changes in their surroundings and to quickly modify their growth and development accordingly [5]. Cold stress, including chilling (0–15 °C) and freezing stress (below 0 °C), is one of the major environmental factors limiting plant growth and development [6]. Cold stress causes complex changes in plants at genetic, biochemical, and physiological levels, while plants have developed a set of defense mechanisms to withstand temperature stress. Cold resistance of plants can be improved by breeding strategies, improved facilities, and the application of exogenous substances. For example, a single nucleotide polymorphism (SNP), SNP2G, in *LOC_Os10g34840* is responsible for conferring cold tolerance at the seedling stage in rice by a genome-wide association study (GWAS) [7]. This research provided genetic resources for breeding cold-tolerant varieties and for studying the molecular basis of cold tolerance in rice. In addition, some evidence indicates that plant tolerance to cold stress is improved by exogenous phytohormones and compounds [8,9,10]. Chang found that the application of 1 µM of melatonin could alleviate the growth inhibition of hulless barley seedlings caused by cold stress [9]. Li found that exogenous DA-6 maintains a high level of ABA content and induces the expression of CBF genes, indicating that DA-6 may participate in the cold response signaling pathway that unravels a mode of action by which plant growth regulators can improve low-temperature tolerance [10]. The application of low concentrations of salicylic acid (SA) to plants could alleviate freezing injuries during low-temperature storage [11]. Recent research shows that exogenous ABA could enhance cold tolerance by increasing the activities of antioxidant enzymes in wheat under cold stress [12]. Many metabolites have been shown to significantly improve cold resistance in plants [9,10,13]. Melatonin serves as an antioxidant to reduce ROS and improves the plant tolerance to cold stress [13]. Treatment with spermidine alleviates the effects of concomitantly applied cold stress in the pollen tubes of Camellia sinensis [14].

As an important non-toxic osmotic protectant, glycine betaine (GB) modulates redox balance, regulates the osmotic potential, and reduces the damage of the cell membrane, which improves plant resistance under various kinds of abiotic stresses [15,16,17]. The accumulation of endogenous GB as well as the application of exogenous GB can improve freezing tolerance during cold domestication in Arabidopsis [18]. Betaine aldehyde dehydrogenase (BADH) is the key gene in the biosynthesis of GB in plants. The heterologous overexpression of *SoBADH* in the transgenic sweet potato improved the content of GB and the tolerance to various abiotic stresses, including salinity, oxidative stress, and cold stresses [19]. Using the RNA interference (RNAi) technique, transgenic rice lines down-regulating *OsBADH1* exhibited a remarkably reduced resistance to NaCl, drought, and cold stresses [20]. Recently, the exogenous application of GB resulted in a greater freezing tolerance in cabbage leaves, as evidenced by lower electrolyte leakage rates (i.e., less membrane damage) and reduced O^2−^ and H_2_O_2_ accumulation [21]. GB accumulation by in vivo expression of the *codA* gene can cause tomato seedlings to be more tolerant of chilling stress than their wild-type counterparts as well as through exogenous applications [22].

The tomato is an important food crop and cash crop that originated in the tropics. Low-temperature conditions cause various injuries to its physiology and development [23]. Since the tomato is a cold-sensitive crop, seed germination occurs at about 21–27 °C [24]. Temperatures below 15 °C will seriously inhibit tomato seed germination [25]. Studies of tomato seed germination have mainly focused on the effects of salt stress [26,27], and there are relatively few reports on the cold tolerance of tomato seed germination. Seed germination is the most critical process in the life cycle of a plant and is regulated by several types of phytohormones and various environmental factors [28]. The seed is susceptible to abiotic (e.g., salt and temperature) and biotic (e.g., seed predators and pathogens) stresses during the germination period. During germination, the root tip of the seed emerges through the seed coat when it is stimulated by the external environment, triggering a series of rapid morphological and physiological changes [29,30]. Therefore, it is important to study the germination of tomato seeds under cold stress. In this study, the effects of exogenous GB on the cold tolerance of tomato seeds were analyzed in terms of germination traits, physiological parameters, and related molecular mechanisms. The aim was to investigate the regulatory role of exogenous GB on cold tolerance during tomato seed germination. The results provide a theoretical basis for exploring the regulatory mechanism underlying the cold tolerance in tomatoes and may guide tomato breeding.

## 2. Results

### 2.1. GB Pretreatment Improves the Germination of Tomato Seeds under Cold Stress

To investigate the effect of GB on tomato seed germination under cold stress, we analyzed various germination parameters under cold stress. As shown in Table 1, the GR of CS seeds was significantly decreased by 52.2% under cold stress compared with the NB group, while pretreatment with 10 mM of GB effectively alleviated the inhibitory effect of cold stress on seed germination. The GR of tomato CB seeds was 2-fold higher than of CS. The GP of the CS group was 35% under cold stress and increased significantly to 75.83% in the CB group. A comparison of the seed GI revealed that GB pretreatment effectively alleviates the inhibitory effect of cold stress on tomato seed germination.

### 2.2. GB Pretreatment Increases Radicle Length in Tomatoes under Cold Stress

**Figure 1:**
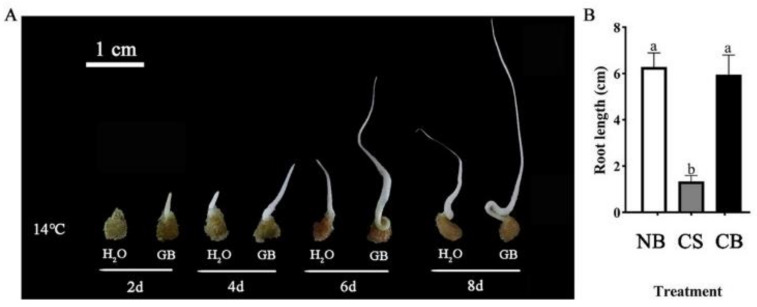
Effects of betaine treatment on tomato seed morphology under low-temperature stress. (**A**) Phenotypes of different treatments at 2, 4, 6, and 8 days. (**B**) Different treatments in seven root lengths. Different lowercase letters indicate significant differences at the 0.05 probability level (*p* < 0.05). The vertical bar represents the mean ± standard error of six repeated calculations. NB: seed germination under monohydrate treatment at room temperature; CS: germination of seeds pretreated with just water under 14 °C cold stress; CB: germination of seeds pretreated in 10 mmol/L of GB under 14 °C cold stress.

A phenotypic analysis of the radicle of germinating seeds was performed on days 2, 4, 6, and 8 post-soaking, as shown in (Figure 1A).

### 2.3. GB Pretreatment Increases the Expression of ROS Scavenging-Related Genes in Tomato Seeds to Improve Cold Tolerance

**Figure 2:**
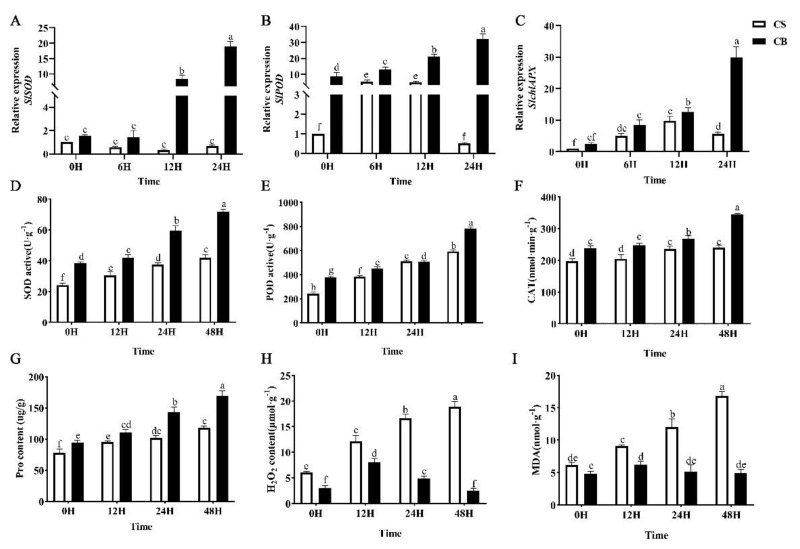
Effects of betaine treatment on the activities of protective enzymes and stress-related growth regulators at 0 h, 12 h, 24 h, and 48 h of tomato seed germination under low-temperature stress. Different lowercase letters indicate significant differences at the 0.05 probability level (*p* < 0.05). (**A**) *SlSOD*, (**B**) *SlPOD*, (**C**) *SlchlAPX*, (**D**) SOD activity, (**E**) POD activity, (**F**) CAT activity, (**G**) Pro content, (**H**) H_2_O_2_ content, (**I**) MDA content. CS: germination of seeds pretreated with just water under 14 °C cold stress; CB: germination of seeds pretreated in 10 mmol/L of GB under 14 °C cold stress.

To clarify the effects of GB on the cold stress resistance of seeds, several physiological indexes related to stress were examined in tomato seeds. Under cold stress, reactive oxygen species (ROS) accumulation was significantly lower in GB-pretreated tomato seeds than in the CS control (Figure 2D–F). Proline (Pro), malondialdehyde (MDA), and H_2_O_2_ play important roles in plants under various abiotic stresses. In this study, we found that the levels of Pro, MDA, and H_2_O_2_ in CB seeds were significantly down-regulated compared with the CS control under cold stress (Figure 2G–I). These results suggest that GB pretreatment induced changes in cold tolerance-related defense enzymes and improved the cold tolerance of the tomato seeds.

In order to explore the molecular mechanism of GB pretreatment that enhances the ROS scavenging ability of tomato seeds, we evaluated the expression levels of key genes involved in ROS production and scavenging under cold stress by qRT-PCR (Figure 2 and Table S1), including *SlchlAPX*, *SlSOD*, and *SlPOD*. The data showed that the expression levels of *SlSOD*, *SlPOD*, and *SlchlAPX*, which encode superoxide dismutase (SOD), peroxidase (POD), and ascorbate peroxidase (APX) in CB seeds were higher than in that of the CS group under cold stress. The expressions of *SlPOD*, *SlSOD,* and *SlchlAPX* in the CB group were significantly up-regulated at 0 h, 12 h, and 24 h, respectively (Figure 2A–C), while the expression of the three genes almost had hardly changed in the CS group. These results suggest that the effect of GB on the cold resistance of tomato seeds may be mediated by ROS scavenging genes.

### 2.4. GB Pretreatment Upregulates α-AMS, β-GAL, and Reduces Starch Content in Tomato Seeds under Cold Stress

**Figure 3:**
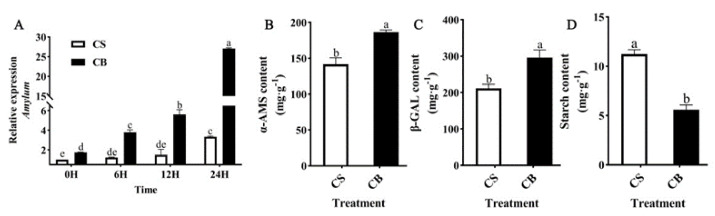
Effects of betaine pretreatment on the expression levels of key genes for starch synthesis in tomato seeds on the seventh day of low-temperature stress: (**A**) amylase related gene expression level, (**B**) α -AMS content, (**C**) β -galactosidase content, (**D**) total starch content in tomato seeds on the seventh day of low-temperature stress. Different lowercase letters indicate significant differences at the 0.05 probability level (*p* < 0.05). CS: germination of seeds pretreated with just water under 14 °C cold stress; CB: germination of seeds pretreated in 10 mmol/L of GB under 14 °C cold stress.

Starch is the main carbohydrate in plant seeds and is catabolized to support the seeds’ germination. To investigate the effect of GB on the total starch content during tomato seed germination under cold stress, various tomato seed germination parameters were determined at seven days. *Amylase*, a key gene related to starch degradation, was significantly up-regulated in the CB group compared to the CS group (Figure 3A). α-amylase is the most important hydrolytic enzyme in early seed germination, which directly determines the seed GR [31,32,33]. β-galactosidase is an enzyme involved in cell wall degradation and provides an essential energy source for seed germination [34,35]. Under cold stress, the α-AMS and β-GAL content of the tomato seeds was significantly increased by 25.05% and 31.36% by GB pretreatment compared with the CS group (Figure 3B,C). The total starch content was significantly down-regulated (Figure 3D). The data indicated that GB pretreatment improved catabolism of stored reserves of starch accumulated during tomato maturation, provided nutrients to the seed germination, and alleviated the inhibitory effect of cold stress on tomato seed germination.

### 2.5. GB Pretreatment Regulates Phytohormone Levels in Tomato Seeds under Cold Stress

**Figure 4:**
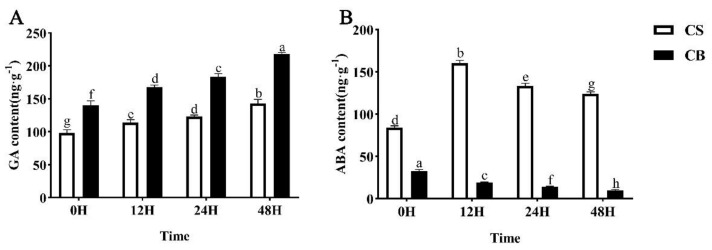
Effects of melatonin on phytohormones under salt stress in cotton seeds. (**A**) Gibberellin (GA) content, (**B**) Abscisic acid content (ABA) content. Different lowercase letters indicate significant differences at the 0.05 probability level (*p* < 0.05) according to Tukey’s multiple range tests. Ver-tical bars indicate the mean calculated for three replications. CS: germination of seeds pretreated with just water under 14 °C cold stress; CB: germination of seeds pretreated in 10 mmol/L of GB under 14 °C cold stress.

The seed germination is mainly controlled by phytohormones, gibberellic acid (GA), and abscisic acid (ABA). GA is the positive plant hormone for seed germination. The synthesis of GA was inhibited in the CS group during seed germination under cold stress. Although the content of GA in the CS group also weakly and gradually increased with the extension of time, the content of GA was still very low compared with the CB group. The GA content in the CB tomato seeds significantly increased by 42.8%, 47.7%, 48.7%, and 52.4% at 0 h, 12 h, 24 h, and 48 h, respectively, compared with the CS group (Figure 4A). These results indicated that GB pretreatment could promote the accumulation of GA in tomato seeds and reduce the damage caused by cold stress.

Under cold stress, tomato seeds showed substantial ABA accumulation, indicating that the synthesis pathway of ABA was activated, or its degradation pathway was inhibited. It was also observed that the ABA content of the CS group peaked at 12 h after germination under cold stress, which was 2.3-fold higher than that in the CB group at the same time (Figure 4B). A gradual decreasing trend of the ABA content in tomato seeds at the germination stage was detected in response to the exogenous application of GB in the CB group. The ABA content in the CB group was reduced by 61.4%, 88.1%, 90.2%, and 92.6% compared to in the CS group at 0, 12, 24, and 48 h of germination, respectively (Figure 4B). The ABA content in tomato seeds pretreated with GB was significantly decreased in the seed germination process compared with that of the control, indicating that GB pretreatment could break tomato seed dormancy and promote seed germination.

### 2.6. GB Pretreatment Regulates the Phytohormone Signaling Genes under Cold Stress

ABA and GA are classical plant hormones that have antagonistic effects on seed germination. The levels of the key genes of the ABA and GA signaling pathways cause different changes during seed germination. To further prove that GB promotes germination by regulating the expression of genes related to hormone signal transduction under cold stress, the expression levels of the GA receptor gene *GID1*; the GA synthetic gene *GA3ox1*; the GA degradation gene *GA2ox*; the ABA receptor genes *SlPLY3* and *SlPLY6*; the ABA synthetic gene *SlABA3*; the negative regulator *SUN24*; and the positive regulators *SlABI3*, *SlABI5*, *SlSnRK2.2*, *SlSnRK2.4*, and *SlSnRK2.5* were analyzed by qRT-PCR (Table S1). 

**Figure 5:**
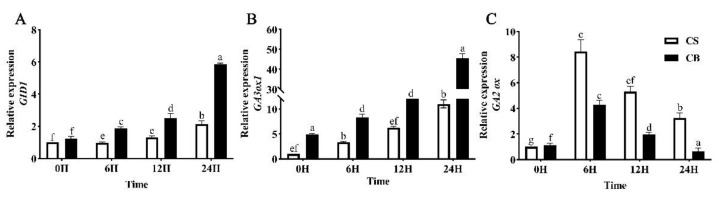
Effects of betaine pretreatment on the expression levels of key genes in GA signal transduction. The content of gibberellin in the neutrons of tomatoes under low-temperature stress was determined by qRT-PCR. (**A**) GID1, (**B**) gA3ox1, (**C**) GA2ox. Vertical bars indicate the mean calculated for three replications. CS: germination of seeds pretreated with just water under 14 °C cold stress; CB: germination of seeds pretreated in 10 mmol/L of GB under 14 °C cold stress. Different lowercase letters indicate significant differences at the 0.05 probability level (*p* < 0.05).

The expression levels of *GIDI* at 12 h and 24 h after GB pretreatment were up-regulated by 64.1% and 76.04% (Figure 5A), respectively, under cold stress. The expression levels of *GA3ox1* were significantly increased at 0, 6, 12, and 24 h, respectively, especially at 24 h (Figure 5B). The expression level of *GA2ox* inactivating bioactive GA was significantly down-regulated in GB-pretreated tomato seeds compared to the CS group (Figure 5C). These results suggested that GB mediated the up-regulated expression of the *GID1* and *GA3ox1* genes and down-regulated the expression of *GA2ox* in the GA signaling pathway under cold stress during seed germination.

**Figure 6:**
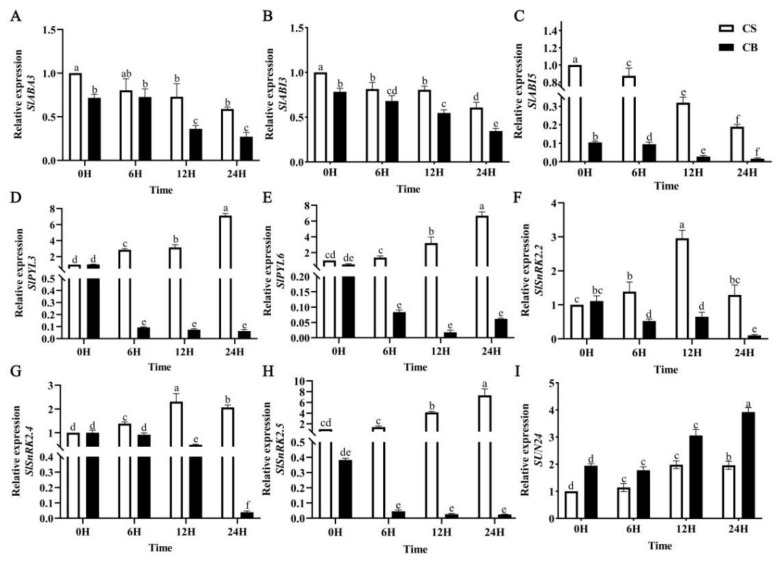
Effects of betaine pretreatment on the expression levels of key genes in ABA signal transduction. The content of abscisic acid in the neutrons of tomatoes under low-temperature stress was determined by qRT-PCR. (**A**) *SlABA3*, (**B**) *SlABI3*, (**C**) *SlABI5*, (**D**) *SlPLY3*, (**E**) *SlPLY6*, (**F**) *SlSnRK2.2*, (**G**) *SlSnRK2.4*, (**H**) *SlSnRK2.5*, (**I**) *SUN24*. Vertical bars indicate the mean ± SEs calculated for three replications. CS: germination of seeds pretreated with just water under 14 °C cold stress; CB: germination of seeds pretreated in 10 mmol/L of GB under 14 °C cold stress. Different lowercase letters indicate significant differences at the 0.05 probability level (*p* < 0.05).

It is well known that the ABA content increases in seeds during the seed maturation and dormancy induction processes to activate the ABA signaling pathway and decreases during seed germination. The expression of key genes in the ABA signaling pathway during seed germination was also analyzed under cold stress. The expression level of *SlABA3* was up-regulated in the CS group and significantly down-regulated in the GB pretreatment group during seed germination under cold stress (Figure 6A). The ABA positive regulators *SlABI3*, *SlABI5*, *SlPLY3*, *SlPLY6*, *SlSnRK2.2*, *SlSnRK2.4*, and *SlSnRK2.5* were significantly down-regulated in the GB-pretreated tomato seeds compared with the CS group under cold stress (Figure 6B–H). The expression levels of *SUN24*, which negatively regulates the ABA signaling pathway, was significantly up-regulated by 37.5% and 47.2%, at 12 h and 24 h in GB-pretreated tomato seeds compared to the CS group (Figure 6I). The above results showed that GB pretreatment down-regulated the positive regulatory genes and up-regulated the negative regulatory genes in the ABA signaling pathway, which can positively modulate seed germination.

## 3. Discussion

Cold stress considerably reduces the seed GP, the seed GI, and antioxidant enzyme activity levels of seedlings and even affects plant photosynthesis in different crops, such as rapeseed (*Brassica napus* L.) [36]. In the present study, exogenous GB could effectively mitigate cold-stress injury in tomato seedlings [37]. Subsequently, they found that exogenous melatonin enhances salt tolerance in cotton seeds by regulating ABA and GA and by mediating the expression of hormone-related genes in plant hormone signal transduction [13]. In this study, we found that appropriate concentrations of exogenous GB could enhance tomato seed germination including GR, GP, GI, and the radicle length of tomato seeds under cold stress. Plants grown in fields are affected by one or more abiotic stresses. Perception of cold stress leads to a variety of responses which include ROS production, antioxidant production, Ca^2+^ release, and activation of multiple transcriptional cascades [38,39]. ROS are used as signaling molecules to activate stress-tolerance mechanisms. Various abiotic stresses can induce excessive ROS in plants and disrupt the balance between the production and scavenging of ROS [40,41]. The relative excessive accumulation of ROS could break the properties of cell membranes and cellular homeostasis [42]. Numerous studies have shown that increasing the levels of ROS scavenging enzymes, such as SOD, CAT, and APX, can improve plant tolerance to abiotic stresses by genetic engineering techniques. Overexpression of *Tamarix albiflonum TaMnSOD* increases drought tolerance in transgenic cotton (*Gossypium hirsutum* L.) by enhancing the development of the root–leaf system and improving the superoxide scavenging capacity [43]. Catalase (CAT) plays a vital role in plant oxidative stress tolerance by scavenging stress induced excess H_2_O_2_. Leucine aminopeptidase 2 (*LAP2*) as a novel *CAT2*-interacting protein can improve the tolerance in conferring plant salt and osmotic stress tolerance [44]. Peroxidase (POD) is a multifunctional enzyme involved in diverse plant physiological processes, including stress tolerance. Lee has shown that the peroxidase gene, *IbLfp,* of sweet potatoes (*Ipomoea batatas* [L.] Lam) is induced by low temperatures, and the tuberous roots of sweet potatoes overexpressing *IbLfp* show improved cold tolerance and lower MDA and H_2_O_2_ content than non-transgenic sweet potato plants under cold stress [45]. Proline as a stress adaptor molecule indicates that proline has a fundamental biological role in stress response. Proline (Pro) as a non-enzymatic antioxidant has a fundamental biological role in stress response [46]. Exogenous melatonin induced plant defense mechanisms by enhancing proline (Pro), total soluble carbohydrates (TSCs), total phenolic compounds (TPC), nutrient (N and P) uptake, and enzymatic and non-enzymatic antioxidants in wheat seedlings under B-toxicity conditions [47]. In this study, we found that exogenous GB enhanced tomato seed germination under typical cold-stress conditions. GB-pretreated tomato seeds showed significantly less ROS accumulation than that in the control by increasing the activity of antioxidant enzyme systems and protecting the integrity of cell membranes under cold stress. Additionally, the Pro content in GB-pretreated tomato seeds was significantly higher than that in the CS group. The expression analysis of ROS related genes further proved the effect of GB on the cold resistance of tomato seeds from the molecular level by qRT-PCR. The data are consistent with the results of previous studies [48,49]. Compared with previous studies, different exogenous plant hormones have basically the same effects on ROS levels of different crops under low-temperature stress, which are all positively regulated, but the main enzymes playing different functions are not the same. Li studied the effect of exogenous melatonin on tea plants under cold stress [50]. The results showed that melatonin treatments can positively up-regulate the gene expression of antioxidant enzyme biosynthesis and effectively alleviate ROS burst. Notably, the upregulation of *CsAPX* was the most significant. In this study, betaine pretreatment could effectively alleviate the ROS outbreak in tomato seeds under low-temperature stress, and *SlPOD* upregulation was the most significant. In short, exogenous GB could cause the global response to cold stress leading to the production of stress response-regulatory proteins or the downstream production of protective proteins or metabolites, which reduce the adverse reaction of the tomato seeds. The endosperm, serving as a storage of the main carbohydrate, induces the hydrolysis of starch in vivo and provides the energy for seed germination and seedling establishment. α-AMS and β-GAL are important hydrolytic enzymes and play important roles in plant ontogenesis [34,51]. Salt stress induced bioactive GA deficiency and inhibited rice seed germination by decreasing α-AMS activity via downregulation of the α-amylase gene expression, which can be rescued by the exogenous bioactive GA application [52]. Exogenous melatonin treatment increased the α-AMS and β-GAL content and decreased the starch content in cotton seeds under salt stress [13]. Our study showed that cold stress leads to decreases in the α-AMS and β-GAL content in tomato seeds, while GB pretreatment can reverse cold-inhibited seed germination by enhancing α-AMS and β-GAL activity.

**Figure 7:**
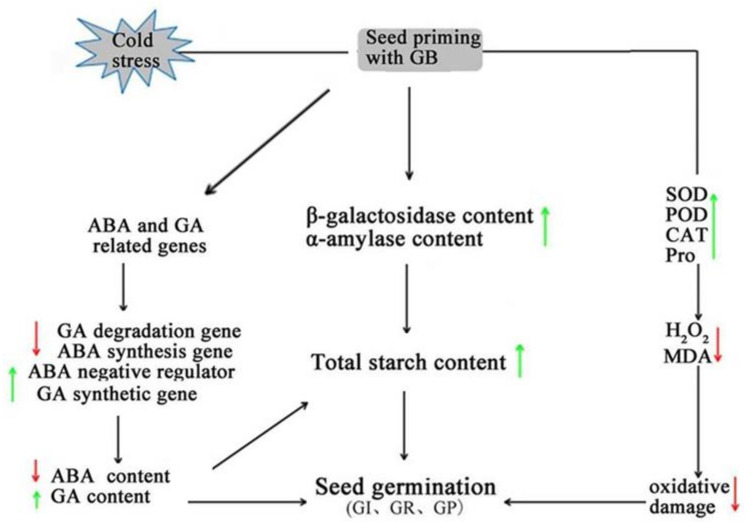
Schematic diagram of the cold tolerance of tomato seeds pretreated with betaine. Low-temperature stress can regulate the expression of plant hormone signal transduction genes, which induce cold tolerance mainly through the expression of plant hormone content and ROS clearance-related genes. The red arrow means down, and the green arrow means up.

Seed germination is a very complex process in which physiological metabolism changes dramatically in a short period of time, including changes of endogenous hormones [53,54]. Phytohormones play critical roles in helping the plants to adapt to adverse environmental conditions. The elaborate hormone signaling networks and their ability to crosstalk make them ideal candidates for mediating defense responses [55]. ABA and GA are the classic phytohormones that regulate plant growth and abiotic stresses and have antagonistic effects on seed germination [28,56]. It is well known that GA promotes seed germination and ABA induces and maintains seed dormancy [57], while the GA and ABA signaling pathways are involved in the stress response of plants [58]. ABA-dependent signaling is one of the major signaling pathways for mediating defense responses under abiotic stress, including low-temperature stress [59]. Interestingly, ABA-insensitive *ABI3*, *ABI4*, and *ABI5* are significantly negative regulators during the seed swelling and early seedling growth stages of Arabidopsis [60]. The plant-specific IQ67-Domain (IQD) proteins are hypothesized to regulate Ca2^+^ signaling and plant development through interactions with calmodulins (CaMs). Bi. found that the tomato IQD gene, *SUN24,* negatively regulates the expression of *SlABI3* and *SlABI5* in germinating seeds [61]. The changes in the GA metabolism and the signal pathway effect the seed germination. The soluble GA receptor, GID1, interacting directly with the DELLA protein in the GA signaling pathway regulates the plant growth and development, including seed germination [13]. The GA receptor gene, *GID1,* and active GA synthesis genes were significantly down-regulated, while the degradation gene was up-regulated under cold stress (Figure 5A–C). The expression levels of ABA receptor genes, synthesis genes, and positive regulators of the signaling pathway were significantly up-regulated, whereas genes encoding negative regulators were down-regulated under cold stress (Figure 6A–I). In contrast, GB pretreatment changed the GA and ABA content or signal pathway in plants and improved the cold tolerance of tomato seeds under cold stress (Figure 4A–B). GB played a key role via two aspects (Figure 7). Firstly, it induced a decrease in the ABA content during seed germination and changed the expression levels of genes in the ABA signaling pathway under cold stress. Secondly, GB induced an increase in GA content; up-regulated the receptor protein gene, *GID1,* and the GA synthesis gene, *GA3ox1*; and down-regulated the degradation gene, *GA2ox,* in GA metabolism. The crosstalk of GA with ABA regulates the balance between seed dormancy and germination; favorable environmental conditions lead to high GA and low ABA levels in seeds, whereas unfavorable conditions cause the reverse ratio [57]. These results showed that exogenous GB attenuated cold-induced injury and promoted the germinability by altering the concentrations of endogenous hormones in tomato seeds. Further, GB is also involved in the regulation of genes and hormone metabolisms for improving seed germination and cold tolerance from molecular mechanisms. In summary, exogenous GB can significantly regulate the balance between GA and ABA in seed dormancy and germination for evading early abiotic stress conditions.

## 4. Materials and Methods

### 4.1. Reagents

All chemicals used in the experiments were of analytical grade. Betaine (Glycine betaine, GB) was purchased from the Sangon Biotech (Shanghai, China).

### 4.2. Plant Material

The tomato (*Solanum lycopersicum*) cultivars “NRP20” tomato seeds used in the experiment were provided by Horticulture Institute of Shanghai Academy of Agricultural Sciences, which is a high-generation inbred line.

### 4.3. Germination Tests

One thousand tomato seeds were sterilized with 75% ethanol for 10 min and rinsed in distilled water four times. They were then divided into three treatment groups: NB (germination of seeds pretreated with just water at room temperature); CS (germination of seeds pretreated with just water under 14 °C cold stress); and CB (germination of seeds pretreated in 10 mmol/L of GB under 14 °C cold stress). Each group was soaked in the different solutions for 24 h and then placed onto an ultra-clean table to dry. One hundred tomato seeds were spread across a total of five Petri dishes (15 cm × 15 cm) with filter paper (Whatman International Ltd., Maidstone, UK) and were cultured at 14 °C and 65% humidity for 7 d in an incubator in the dark. Seed germination potential (GP) and the germination rate (GR) were recorded. GP and GR were recorded on the third and seventh day, respectively. All phenotypic measurements included six independent biological repeats. Seeds were considered to be germinated when the total radicle and hypocotyl length exceeded half the length of the seed. Germination of seeds was recorded daily. The numbers of germinated seeds on day 3 and 7 after initiation were the germination potential (GP) and the germination rate (GR), respectively [62,63]. Germination index (GI) was calculated using the method developed by Wang [64].

### 4.4. Morphological Observation and Determination of Hypocotyl Length

Tomato seed morphology was observed at 2, 4, 6, and 8 d. The hypocotyl length of 50 tomato seeds was measured on day 7 with a Vernier caliper. Six independent biological repeats were measured.

### 4.5. Determination of α-Amylase (EC3.3.1.1),β-Galactosidase (EC3.2.1.23), and Starch Content

The activity of α-amylase (α-AMS) and β-galactosidase (β-GAL) in the tomato seeds was measured on the 7th day of tomato seed germination according to the manufacturer’s protocol using the kit from the Shanghai MLBIO Biotechnology Co., Ltd. (Shanghai, China). The starch content of cotton seeds was determined on the 7th day of germination with the starch kit provided by Beijing Box Biotechnology Co., Ltd. (Beijing, China), including 6 independent biological repeats.

### 4.6. Extraction and Assay of Phytohormone, ABA, and Gibberellin (GA3)

The content of ABA and GA at different time points (0, 12, 24, and 48 h) of seed germination was determined by enzyme-linked immunosorbent assay (ELISA) provided by Shanghai MLBIO Biotechnology Co., Ltd., including six independent biological repeats.

### 4.7. RNA Isolation and Quantitative Real-Time PCR (qRT-PCR) Analysis

According to the manufacturer’s (Hangzhou Borui Technology Company) plan the Biospin Plant Total RNA Extraction Kit was used to extract the total RNA of the tomato seeds at different time points (0, 6, 12, and 24 h). The RNA in each sample was reverse transcribed using the total RNA as the template, and the specific method was conducted according to the HiScript II One Step RT-PCR Kit to obtain cDNA products. The qRT-PCR analysis was performed using reverse transcript cDNA as the template and the eIF gene as the reference gene. The ratio and procedure settings of each reaction solution were configured and set according to the Hieff UNICON^®^ Universal Blue qPCR SYBR Green Master Mix reagent instructions from Yeasen Biotechnology (Shanghai) Co., Ltd. to calculate the expressed 2-∆ Ct value. Each reaction was performed in triplicate.

### 4.8. Statistical Analysis

Data was collated using Excel and analyzed for statistics and significance using SPSS software 22.0 (IBM, Armonk, NY, USA) with a *p*-value of 0.05, indicating a significant difference. Drawing was conducted using the GraphPad Prism 8 (GraphPad Software, San Diego, CA, USA), and phenotype observations were photographed with a Canon camera.

## 5. Conclusions

Exposure to cold temperatures in the immediate environment alters various biochemical processes, which can delay or inhibit the germination processes of seeds. We comprehensively evaluated the effects of GB on the germination of economically valuable tomato seeds under cold stress. Exogenous GB can reduce seed damage under cold stress by altering levels of endogenous oxidants, metabolites, and phytohormones, thereby promoting seed germination. Therefore, it is reasonable to assume that multiple stress signals could converge on the same intracellular signaling components to affect a response. This is the first analysis of the effect of GB on tomato seed germination under cold stress, providing a basis for tomato cultivation technology and engineering. Future studies should further explore the mechanism by which GB protects plants against cold stress and promotes plant growth.

## Figures and Tables

**Table 1:** Effect of different treatment on the GR, GP, and GI of tomato seeds.

Treatment	Germination Rate (%)	Germination Potential (%)	Germination Index
NB	95.53 ± 4.56 ^a^	91.67 ± 3.64 ^a^	64.48 ± 1.95 ^a^
CS	37.6 ± 3.86 ^c^	35 ± 2.04 ^c^	10.85 ± 0.53 ^c^
CB	78.5 ± 2.98 ^b^	75.83 ± 3.12 ^b^	44.48 ± 1.95 ^b^

NB: seed germination under monohydrate treatment at room temperature; CS: germination of seeds pretreated with just water under 14 °C cold stress; CB: germination of seeds pretreated in 10 mmol/L of GB under 14 °C cold stress. Different lowercase letters indicate significant differences at the 0.05 probability level (*p* < 0.05).

## Supplementary Materials

The following supporting information can be downloaded at: https://www.mdpi.com/article/10.3390/ijms231810474/s1.
