# Supplementary material for: Effect of Exogenous Glycine Betaine on the Germination of Tomato Seeds under Cold Stress
Source: Int J Mol Sci. 2022 Sep 9;23(18):10474. doi: 10.3390/ijms231810474 (PMC9502054; doi:10.3390/ijms231810474)
Supplement: Supplementary file 1 [file ijms-23-10474-s001.zip › ijms-1846483-supplementary.pdf]

Table S1. Primers used for q-RT-PCR.

**Gene Name**	**Forward (5'-3')**	**Reverse (5'-3')**
Alpha-amylase (Solyc03g095710)	ACGGTGTTGTTTCAGGGGTT	ACTCCAGCATGAGCCAAGTC
SlSOD (Solyc01g067740)	ACATACAAAAATGGTGAAGGCC	AGGATTGTAATGTGGTCCTGTT
SlPOD (Solyc11g018800)	GGTCAACGGATAGCATTGTTAC	TTTATAGCGCCACAAACAGTTC
SlChlAPX (Solyc06g005160)	GACAATTCATACTTCACGGAGC	CATAGTCAGCAAAGAAGGCATC
SlPYL3 (Solyc01g095700)	ATCACAGGCTAAAGAACTATTCTTCGG	ACCAGTTCACACTGACGGGTAGAAC
SlPYL6 (Solyc06g050500)	GCACAGAACTTGGCTAAGACGTCAA	CAAGAACCAACCTTACAGTCCACGA
SlSnRK2.2 (Solyc05g056550)	CTCCAGTTTCCCGTTCAGTCTCAG	CTGCGAGCAAGCAAGATAATTTACG
SlSnRK2.4 (Solyc02g090390)	TTCCAGCGGACTTAATAGACGATAGG	TCACAAGCTTGGCATGCACTCAC
SlSnRK2.5 (Solyc11g010310)	GAGATAGAGACCAGTGGGGAATTCG	AACCCCTTCGCTCATCTCTATCTCA
SUN24 (Solyc08g083240)	GGGCTTTCAGGTCACCAACT	TCTGATCCATGTCTCCGTGA
SlABI3 (Solyc06g083590)	ATCCAAAACTCTGCCCCTTT	GTAGCACAAGTGCCCAACCT
SlABI5 (Solyc09g009490)	GGGAAATGTTTCGTTGGAGA	TGTATGTTGCACCCGTTGTT
GA3ox1 (Solyc06g066820	GCCTCCTTCCACAGATCCAG	CAAACCGGAGGCTTCCTTGA
GA2ox1 (Solyc05g053340)	CACCGACGATGCCAAGAA	CCACGACGGAGAAGACACTG
GID1 (Solyc09g074270)	CGCCGTAAGGAACAGAACAT	CCGCAGCGACGCAGTA
SlABA3 (Solyc07g066480)	ATGAACATCGAATCGGAGAAAGAG	ACCGTATCGTTTAAGCGCTTAAAT
